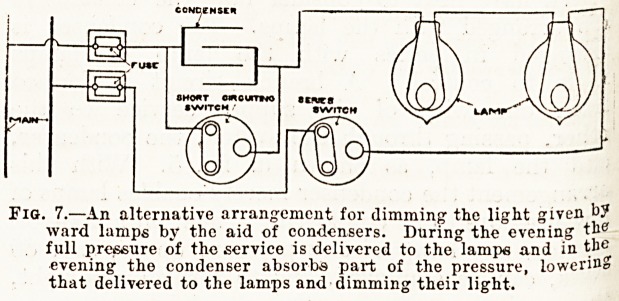# The Lighting of Wards.—II

**Published:** 1912-05-18

**Authors:** 


### PRACTICAL POINTS.

(Criticism and Suggestions Invited.)

### The Lighting of Wards.—II.

We have next to consider how the condensers are arranged with the lamps. The condenser is arranged in series with the lamp or lamps it is to control. A branch circuit is formed from one wire of the supply service to the other, passing through the switch, the condenser, and the lamp, as shown in fig. 5. With this arrangement the condenser merely enables lamps of low pressure to be employed. Thus, on a 200-volt service, any lamp of any pressure up to, say, 80 volts, can be employed, and to furnish any light, the condenser using up the remaining pressure. Thus, if a 10-volt lamp is employed, the condenser accounts for the 190 volts; if a 25-volt lamp is employed, the condenser accounts for the 175 volts.

An extension of this arrangement is shown in fig. 6, in which two or more lamps are connected in series with a condenser. A branch circuit is formed as before, including the condenser and the

Fig. 5.—A single incandescent lamp in circuit with an electric condenser.

lamps; but in this case, in addition to the switch cutting off the supply of current to both condensers and lamps, there is a short-circuiting switch to each lamp. For reasons which there is not space to enter into here, 40 per cent. of the service pressure is found to be the economic limit up to which lamps may be employed with condensers in this arrangement; or, to put it in another way, 60 per cent. of the pressure is the economic limit of the amount which the condenser can be made to account for.

Where two or more lamps are arranged in series with the condenser, as shown in fig. 6, they may either be of the same candle-power or of different candle-powers, and they may be arranged to work with equal pressures or with different pressures. Thus, with a 200-volt service, 80 volts may be used up in lamps, of which there may be, say, eight lamps, each taking 10 volts, and each furnishing, say, 10 candle-power, 25 candle-power, or whatever may be desired; or, the pressures and candle-powers may be varied. For ward lighting, for instance, there may be one or two lamps of high candle-power in the centre of the ward, lamps of smaller candle-power at the sides of the ward, and one or two of very low candle-power where convenient for night service. The high candle-power lamps would in that case work with higher pressures than those of smaller candle-power, and the very low candle-power lamps, those designed for night work, would work with the lowest pressure for which lamps can be made.

Each lamp, as explained, would be short-circuited, its terminals bridged across when it was

Fig. 6.—Arrangement for supplying lamps of different candle-powers from the same supply service with a condenser in series. In the above the service is supposed to be at 200 volts and the condenser taking 121 volts, a 50 c.-p. lamp 50 volts, a 16 c.-p. lamp 16 volts, and one or more 3 c.-p. lamps 3 volts. During the evening, when light is wanted, the 3 c.-p. lamps are cut out by the short circuiting switch, and at night the 16 c.-p. and 50 c.-p. are cut out in the same way.

to be turned out, and there would be a controlling switch for the whole of the series of lamps and condenser, which would be brought into service when no light whatever is required. In fig. 6 one 50 candle-power lamp taking 50 volts is shown, one 16 candle-power taking 16 volts, and one 3 candle-power taking 3 volts. The condensers are calculated for a given current, and to answer for a certain difference in pressure. That is what causes the economic limit of 40 per cent. in lamps, or 60 per cent. in condenser; the difficulty of constructing a condenser to utilise the same current as the lamps, and to maintain the pressure in the different lamps at the normal when varying numbers of them are burning, if the lamps take a higher percentage of the total pressure. There is a slight variation in the current as lamps are turned in or out, but up to 40 per cent. of the pressure it is negligible.

It will be seen that with this arrangement the greatest possible economy is obtained. The condenser practically uses no current; there is no waste of current due to a resistance being heated, and, in addition, economies in renewals of lamps and in the current taken by the lamps are obtained by the ability to use lower voltage and stronger filament lamps.

There is an alternative arrangement for dimming the light given by the ward lamps at night, by the aid of condensers, on somewhat similar lines to the arrangements described above, in which lamps are put in series or in parallel. The branch circuit supplying any number of lamps is made to include a condenser, which is fitted with a short-circuiting switch, in place of the lamps having a short-circuiting switch. There is the usual switch for opening and closing the branch circuit. During the evening the full pressure of the service is delivered to the lamps, and they furnish their full light, the condenser being short-circuited. At night the short-circuiting switch of the condenser is opened, and the pressure delivered to the lamps is lowered by the amount of pressure absorbed by the condenser.

Fig. 7.—An alternative arrangement for dimming the light given by ward lamps by the aid of condensers. During the evening the full pressure of the service is delivered to the lamps and in the evening the condenser absorbs part of the pressure, lowering that delivered to the lamps and dimming their light.

This arrangement is not wasteful as those described in the earlier part of the article are, because the condenser does not waste current, it merely absorbs pressure. The arrangement is shown in fig. 7.

## Figures and Tables

**Fig. 5. f1:**
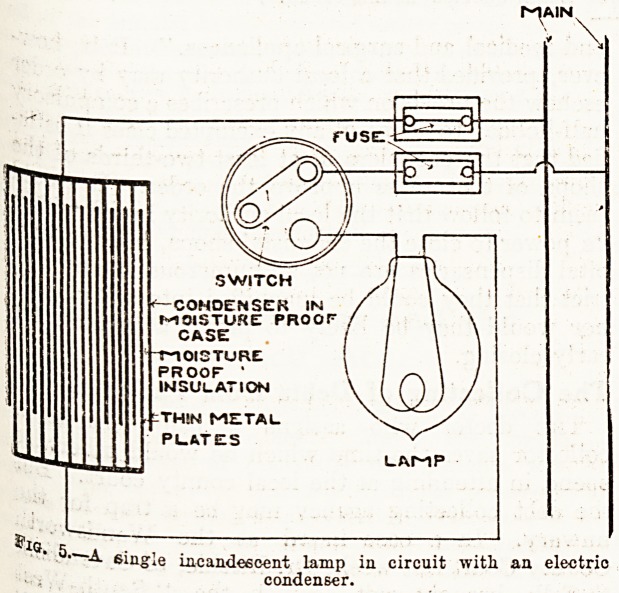


**Fig. 6. f2:**
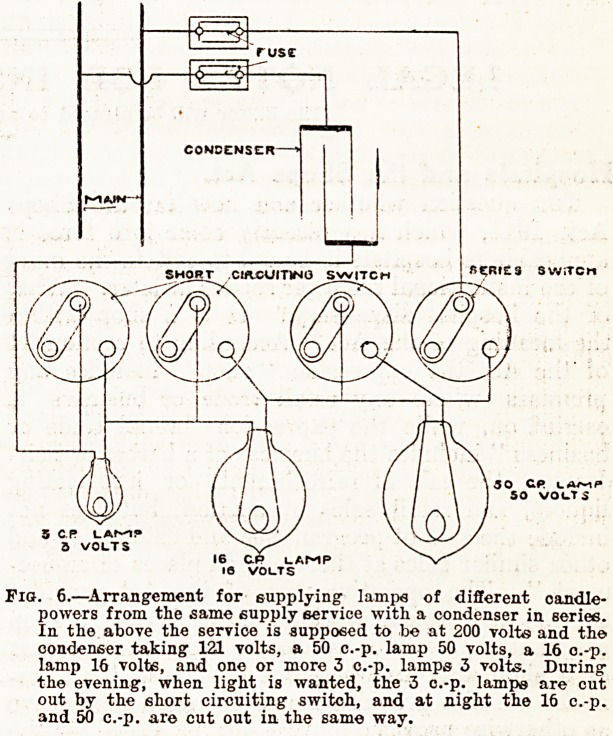


**Fig. 7. f3:**